# Endoscopic drainage for the treatment of intramural colonic abscess

**DOI:** 10.1055/a-2612-3394

**Published:** 2025-06-26

**Authors:** Yuki Amioka, Satoshi Masuda, Hidenori Tanaka, Ken Yamashita, Yoshihiro Kishida, Toshio Kuwai, Shiro Oka

**Affiliations:** 1 68272 Department of Gastroenterology, Hiroshima University Hospital, Hiroshima, Japan

A 70-year-old man presented to our hospital with abdominal pain and vomiting. Contrast-enhanced computed tomography (CT) revealed a capsulated abscess adjacent to the descending colon causing intestinal obstruction ( Fig. 1 ). A retrospective review of a CT scan from 2 years prior identified a linear hyperdense structure resembling a fish bone penetrating the intestinal wall at the same site, which was suspected to be the cause of the abscess.

**Fig. 1:**
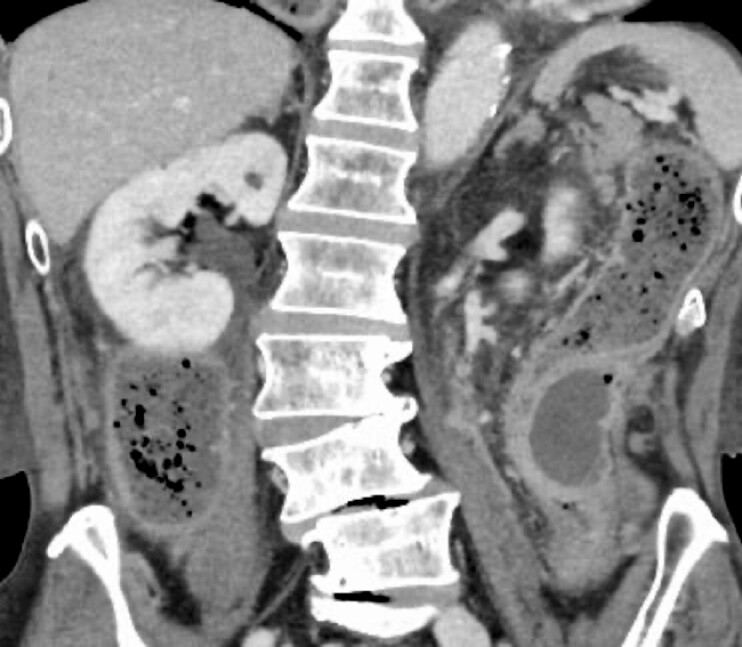
A capsulated abscess causing intestinal obstruction.

Colonoscopy revealed a submucosal tumor-like elevation corresponding to the abscess ( Fig. 2 **a** ), with luminal obstruction preventing the passage of the scope. Endoscopic ultrasonography (EUS) demonstrated a homogeneous hypoechoic lesion with internal hyperechoic foci, predominantly in the submucosa, with a preserved muscularis propria ( Fig. 2 > **b** ). Given the intramural nature of the abscess, percutaneous CT-guided drainage posed a risk of perforation or fistula formation. Therefore, endoscopic drainage was attempted via colonoscopy ( Video 1 ).

**Fig. 2:**
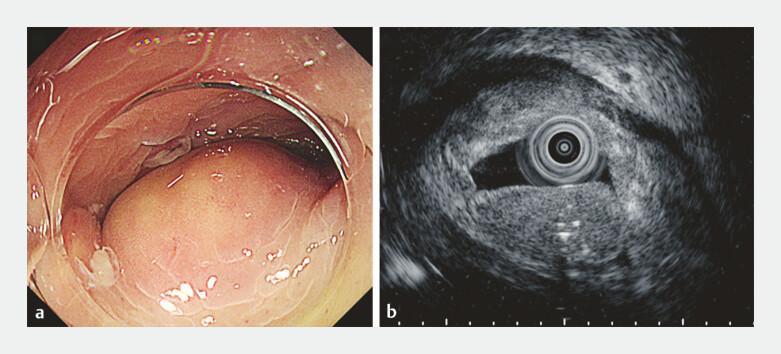
**a** Colonoscopy revealed a submucosal tumor-like elevation. **b** Endoscopic ultrasonography demonstrated a homogeneous hypoechoic lesion with internal hyperechoic foci.

By utilizing EUS and CT for precise diagnosis, we safely performed minimally invasive endoscopic drainage for the treatment of intramural colonic abscess, achieving symptom resolution.Video 1

A 10 mm mucosal incision was made using DualKnife J (Olympus Medical Systems), leading to purulent evacuation ( Fig. 3 ). Sufficient drainage via suction and mucosal compression allowed the scope to pass ( Fig. 4 ). The symptoms resolved immediately, and the patient was discharged 9 days later. Follow-up CT 2 months later confirmed the resolution of the abscess with minor residual intramural air ( Fig. 5 ).

**Fig. 3:**
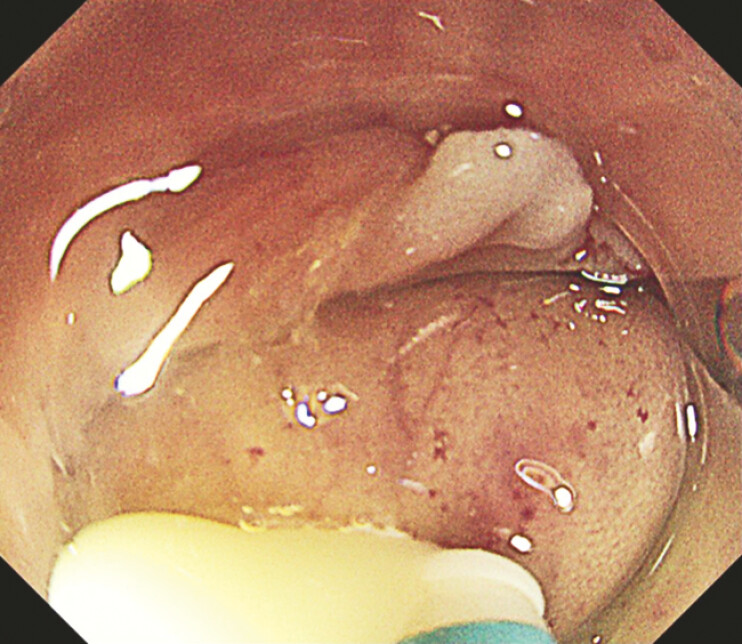
A 10 mm mucosal incision was made using DualKnife J, leading to purulent evacuation.

**Fig. 4:**
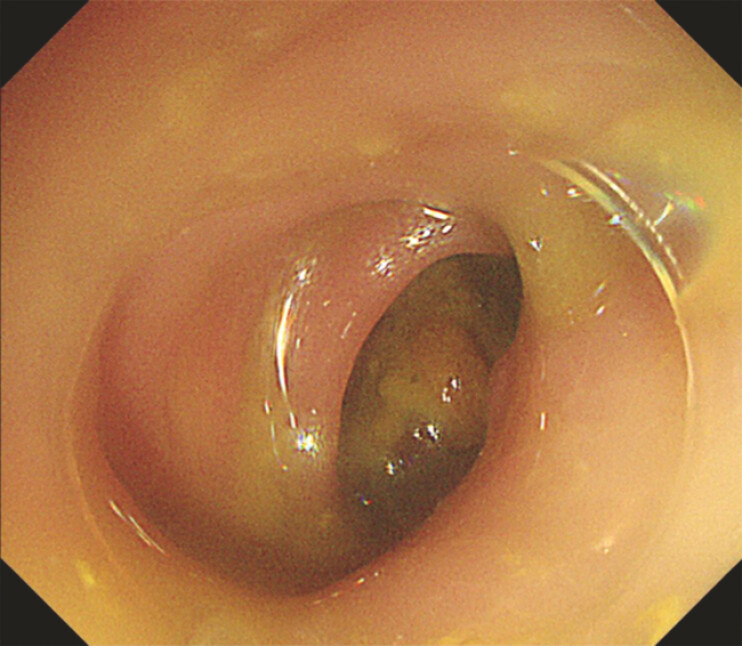
Sufficient drainage via suction and mucosal compression allowed the scope to pass.

**Fig. 5:**
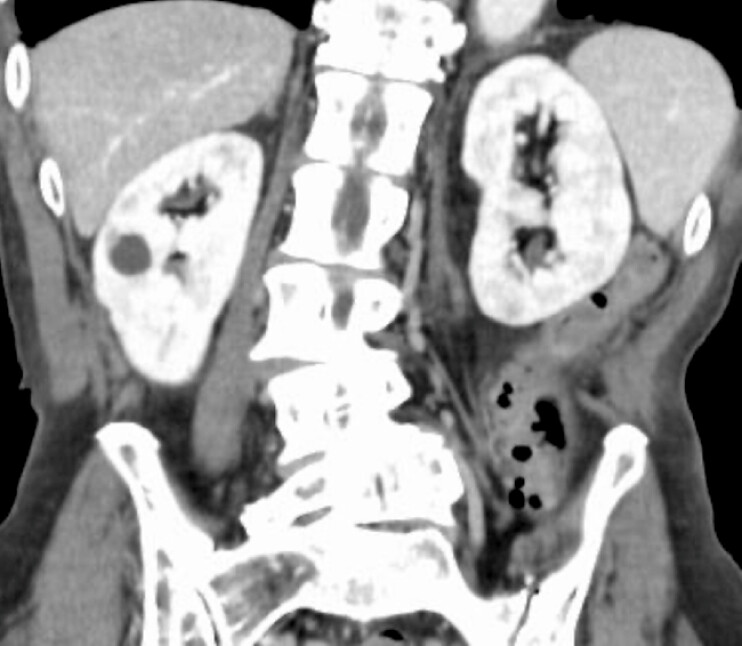
Follow-up CT 2 months later confirmed the resolution of the abscess with minor residual intramural air. Abbreviation: CT, computed tomography.

Colonic intramural abscesses are rare, and to our knowledge, there have been no previous reports of endoscopic incision and drainage for relief of obstruction, although several cases have been reported in the esophagus and stomach 1 2 3 . Recently, EUS-guided drainage has proven useful for extramural abdominal abscesses 4 . In this case of intramural abscess, we performed minimally invasive endoscopic drainage via mucosal incision alone. Procedural safety was ensured through precise diagnosis with EUS and CT. This case highlights the potential of endoscopic treatment as a viable alternative to surgical or percutaneous drainage for colonic intramural abscesses.

Endoscopy_UCTN_Code_TTT_1AS_2AJ

## Notes

Endoscopy E-Videos https://eref.thieme.de/e-videos
*E-Videos* is an open access online section of the journal *Endoscopy* , reporting on interesting cases and new techniques in gastroenterological endoscopy. All papers include a high-quality video and are published with a Creative Commons CC-BY license. Endoscopy E-Videos qualify for HINARI discounts and waivers and eligibility is automatically checked during the submission process. We grant 100% waivers to articles whose corresponding authors are based in Group A countries and 50% waivers to those who are based in Group B countries as classified by Research4Life (see: https://www.research4life.org/access/eligibility/ ). This section has its own submission website at https://mc.manuscriptcentral.com/e-videos .
